# Quantified Interfacial Affinity of Hydrogen Sulfate and Sulfate Speciation in Model Marine Aerosol Particles

**DOI:** 10.1021/acs.jpca.6c04034

**Published:** 2026-09-04

**Authors:** Leslie M. Ritchie, Madison B. Lester, Arun K. Sharma, Joshua D. Patterson

**Affiliations:** † Department of Biology, Chemistry, and Environmental Science, Christopher Newport University, Newport News, Virginia 23606, United States; ‡ Department of Biology, Agriculture, and Chemistry, California State University Monterey Bay, Seaside, California 93955, United States

## Abstract

Heterogeneous reactions occurring in atmospheric aerosols are crucial components of multiple chemical reaction cycles. Sulfate species exert substantial influence on many heterogeneous reaction mechanisms due to their abundance and speciation. Despite the importance of sulfate species on heterogeneous reactions, quantitative descriptions of interfacial affinity and sulfate species partitioning at interfaces remain incomplete. Here, we quantify the interfacial properties of HSO_4_^–^, using cetyltrimethylammonium hydrogen sulfate (CTAHS) reverse micelles (RMs), as model marine aerosol particles. Infrared spectra (IR) of CTAHS RMs were collected and analyzed using multivariate curve resolution-alternating least-squares (MCR-ALS). MCR-ALS analysis reveals that four distinct species contribute to the observed IR spectra, which we assign as CTA^+^-HSO_4_^–^ contact pairs (CPs), interfacial HSO_4_^–^, core HSO_4_^–^, and core SO_4_^2–^. Computational studies reveal that the CTA^+^-HSO_4_^–^ CPs exist in two distinct orientations at the interface. Spectral contributions from each chemical species are used to determine the quantitative interfacial affinity (χ_Int._) of HSO_4_^–^. Our observations indicate that the cationic charge of the interface alters the ionic distribution of sulfate species compared to air–water interfaces, placing H_3_O^+^ below HSO_4_^–^.

## Graphical abstract

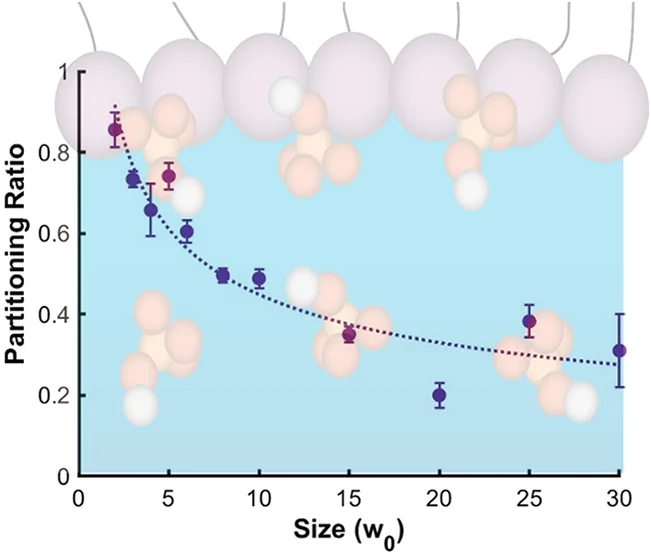

## Introduction

Heterogeneous or mixed-phase reactions have long been considered vital components of numerous atmospheric reaction cycles, from Antarctic ozone to acid rain.^1–19^ Despite the importance of these reactions, the accurate prediction of heterogeneous reactions faces considerable challenges. These complex reactions include multiple reaction steps, occurring over multiple time scales, and involve compositionally diverse reactants in the form of aerosols. Because of these challenges, deviations as large as 100% ^14,18,20,21^ have been observed between predicted and measured wintertime particulate NO_3_^–^ concentrations, attributed to inaccurate heterogeneous reaction rates or incomplete mechanistic descriptions.

While numerous heterogeneous reaction mechanisms include ionic reactants in or on the surface of aerosol particles, few ionic species exert a larger impact on heterogeneous reactions in the atmosphere than sulfate. Sulfate is the most common inorganic species in the upper troposphere, estimated to be about 90% of all inorganic species, and the third most abundant ionic species, by mass, in seawater.^22^ Capable of existing in multiple protonation states, SO_4_^2–^, HSO_4_^–^, and H_2_SO_4_, sulfate species can influence the uptake of water and ammonia, modulate aerosol growth rates, facilitate the formation of polar stratospheric clouds, and regulate aerosol pH, all of which can alter heterogeneous reaction rates.^2,15,23–25^ As such, sulfate species have frequently been used as aerosol substrates for laboratory studies of heterogeneous reactions, including the reactive uptake of dinitrogen pentoxide (N_2_O_5_), an important reservoir species of anthropogenic nitric oxides.^1,2,5,6,26^ While not directly involved in the hydrolysis reaction of N_2_O_5_, by examining reactive uptake as a function of particle radius, Gaston and Thornton determined that sulfate speciation influenced the pseudo-first-order rate for N_2_O_5_ hydrolysis, with SO_4_^2–^ yielding a larger rate constant than HSO_4_^–^.^1^ As hydrolysis of N_2_O_5_ is estimated to account for 25–41% of all removal of N_2_O_5_ in the troposphere,^7–12^ molecular-scale speciation of sulfates could have terrestrial-scale impacts on atmospheric chemistry.

The large impact of sulfate species on heterogeneous reactions throughout the atmosphere has motivated numerous studies targeting the distribution of sulfate species at the interfaces. Spectroscopic experiments and molecular dynamics (MD) simulations have been combined to develop a framework for describing the distribution of sulfate species at air–water interfaces.^27–32^ HSO_4_^–^ has a stronger relative interfacial affinity than SO_4_^2–^, which is often excluded or buried deep within the interfacial region. Cations typically localize between HSO_4_^–^ and SO_4_^2–^ within the interface, except for H_3_O^+^, which exhibits a preference for the interface that exceeds that of HSO_4_^–^, localizing at the surface. This framework has been vital in explaining the observed substrate dependence of the N_2_O_5_ uptake rates. The lower relative interfacial affinity of SO_4_^2–^ creates greater availability of H_2_O at the aerosol surface, increasing the pseudo-first-order rate constant for N_2_O_5_ hydrolysis relative to HSO_4_^–^.^1^

Despite the considerable advances made in understanding the distribution of sulfate species at interfaces, quantitative assessments of interfacial affinity and sulfate species partitioning remain elusive. These parameters are critical to bridging the gap between the molecular-scale details provided by interfacial studies and the bulk aerosol properties used to model heterogeneous reaction rates. Here we use cetyltrimethylammonium hydrogen sulfate (CTAHS) reverse micelles (RMs) as proxies for marine or sea spray aerosol particles to examine the speciation of sulfates and quantify the affinity of HSO_4_^–^ for the interface. Vibrational spectroscopy methods have been frequently used to examine molecular species at interfaces, and the combination of vibrational spectroscopy with RMs has proven to be an effective strategy for isolating the vibrational signatures of interfacial species, examining interfacial-induced structural modifications, and probing local dynamic information.^33–43^ RMs in solution possess the same structural morphology as sea spray aerosols^3,33^ and can be readily tuned by varying the molar ratio of water to surfactant, *w*_0_ = [H_2_O]/[surfactant], thereby adjusting the proportion of core and interfacial water within the aqueous interior. The RM interfacial region is host to a myriad of chemical species, often with overlapping vibrational spectra, presenting a considerable obstacle toward quantifying the populations of the individual chemical species. Multivariate curve resolution–alternate least-squares (MCR-ALS) analysis allows for the deconvolution of complex spectra to isolate both pure spectral identities and determine the contributions of each unique species to the observed spectra.^44,45^ MCR-ALS analysis of collected infrared (IR) absorption spectra of CTAHS RMs reveals the presence of four spectral distinct sulfate species: CTA^+^-HSO_4_^–^ contact pair (CP), interfacial HSO_4_^–^, core HSO_4_^–^, and core SO_4_^2–^. Computational studies of CTA^+^ and HSO_4_^–^ ion pairs indicate the presence of two orientational subpopulations within the interface. By examining the partitioning of interfacial HSO_4_^–^ species as a function of *w*_0_, we quantify the preference of HSO_4_^–^ for the interface and propose an ionic distribution within the interface that favors HSO_4_^–^ species over H_3_O^+^ and SO_4_^2–^.

## Methods

### Materials

CTAHS (Sigma-Aldrich, 99%) and CTAB (Sigma-Aldrich, 95%) were dissolved in methanol and then dried under vacuum to remove the methanol and residual water. The dried surfactants were stored over P_2_O_5_ under vacuum prior to use. Chloroform (ACROS, 99.8%) and isooctane (Fisher, 99.7%) were used without additional purification. Deionized water (18 MΩ) was used for aqueous sample preparation.

### Reverse Micelle Preparation

Surfactant stock solutions of 0.25 M CTAHS and CTAB were prepared in 5:1 chloroform/isooctane solvent mixtures. RMs were prepared by adding appropriate amounts of deionized water and solvent to the surfactant solutions based on the desired *w*_0_ ratio and fixing the surfactant concentration at 0.1125 M. RM samples were vortexed and sonicated prior to use.

### IR Characterization

IR absorption spectra were collected on a Shimadzu IRTracer-100 FT-IR. RM samples were held in a 0.25 μm path length cell equipped with CaF_2_ windows. IR absorption spectra were collected over the range of 4000–400 cm^–1^ at a 1 cm^–1^ resolution and based on an average of 48 scans. The spectra were baselined, and then CTAB RM spectra were subtracted from CTAHS RM spectra of equivalent *w*_0_’s to remove spectral contributions from the solvent, water, and surfactant. The resulting spectra were truncated to 1100–1000 cm^–1^, baselined, and normalized to isolate the vibrational signature of HSO_4_^–^ and SO_4_^2–^.

### MCR-ALS Analysis Performed Using MCR-ALS GUI 2.0 ^44,45^

A total of 66 spectra were compiled into a single spectral matrix. The number of components was set at 4 based on the results of the initial singular-value decomposition of the spectral matrix. Initial spectral estimation was conducted using the purest variable detection. A non-negativity constraint was applied to the spectral analysis, while non-negativity and closure constraints were applied to the concentration analysis. The concentration closure constraint was set equal to 1, to allow for sulfate speciation to change without impacting the total sulfate concentration.

### Theoretical Methods

Computational strategies for examining CTA^+^-anion complexes have been described elsewhere.^46^ Briefly, Møller–Plesset perturbation theory^47,48^ calculations with the 6-311+G­(d,p)^49^ basis sets were applied for the initial optimization of the bare HSO_4_^–^ ion in Gaussian16. Our own N-layered Integrated molecular Orbital and Molecular mechanics (ONIOM)^50–52^ two-layer procedure was used to optimize the CTA^+^ cation with a low- and high-layer level of theory of semiempirical/PM6 ^53^ and MP2/6-311+G­(d,p), respectively. The ONIOM method has been successfully used in previous comparative studies as a two-layer quantum mechanics (QM)/molecular mechanics (MM) method and uniquely combined QM and MM methods have been extended to multiple layers for modeling systems in solvated conditions.^54–56^ These initial geometries were used for optimized geometry searches for CTA^+^-HSO_4_^–^. Integral Equation Formalism Polarizable Continuum Model (IEFPCM), an implicit solvation model, was used to approximate bulk solvent polarization effects during geometry optimization and frequency calculations.^57–59^ To mimic ion-pair separation, structures were generated by increasing and decreasing the distance of the HSO_4_^–^ from CTA^+^ in steps of 0.5 Å and 0.25 Å from the optimal geometry distance, respectively. Frequency calculations were performed at each displaced geometry without subsequent reoptimization. The displaced structures are therefore nonstationary points on the potential energy surface, and the harmonic analysis is not intended to characterize equilibrium vibrational structure. The calculations were instead used to follow the response of localized, high-frequency diagnostic bands in alignment with experimental data (υ_1_(*a*_1_) symmetric SO_3_ stretch mode of HSO_4_^–^), as a function of the CTA^+^-HSO_4_^–^ separation. No empirical vibrational frequency scaling factor was applied, because the analysis focused on relative frequency shifts obtained using the same computational treatment rather than quantitative reproduction of experimental band positions. Low-frequency and intermolecular modes were excluded from this analysis, and thermochemical quantities were not evaluated.

## Results and Discussion

The normalized IR absorption spectra of HSO_4_^–^ within CTAHS RMs at various *w*_0_ ratios are shown in Figure 1. The spectra are dominated by a central peak located near 1050 cm^–1^. As *w*_0_ increases, this peak undergoes a 3 cm^–1^ blue-shift. The variation in *w*_0_ also reveals two additional spectral features: a shoulder at 1060 cm^–1^, observed in the *w*_0_ = 2 RM spectrum, and a broad peak near 1100 cm^–1^ that grows in as *w*_0_ increases. The spectral evolution suggests that HSO_4_^–^ undergoes either substantial changes in IR absorption properties induced by variation in the local environment or converts to other molecular species as *w*_0_ increases.

**Figure 1:**
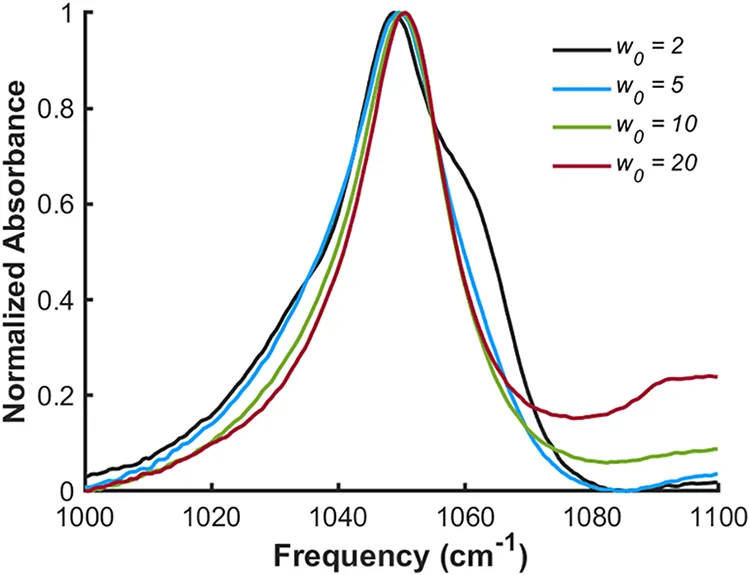
Normalized IR absorption spectra in CTAHS RMs for *w*_0_ = 2 (black line), *w*_0_ = 5 (blue line), *w*_0_ = 10 (green line), and *w*_0_ = 20 (red line).

To determine the sources of the observed spectral evolution, the normalized IR absorption spectra were analyzed using MCR-ALS.^44,45^ MCR-ALS analysis identified 4 unique components that contribute to the observed spectra. Fit convergence for the entire spectral matrix was achieved following 8 iterations with a corresponding *r* ^2^ = 99.852. The associated pure spectra of the 4 components are shown in Figure 2. Representative fits of experimental spectra using pure spectra are shown in Figures S1–S4. The deconvolved pure spectra consist of two individual peaks located at 1049 and 1051 cm^–1^, a broad peak at 1090 cm^–1^, and a bimodal spectrum with peaks located at 1047 and 1061 cm^–1^. Previous studies of HSO_4_^–^ in aqueous solution have observed single peaks in the range of 1051–1052 cm^–1^, assigned to the symmetric SO_3_ stretch of HSO_4_^–^ (υ_1_(*a*_1_)*v*_s_SO_3_) vibrational mode.^60,61^ Consistent with these observations, the peak at 1051 cm^–1^ is attributed to HSO_4_^–^ located in the aqueous core of the RM structures. Studies of interfacial-core partitioning in RMs have frequently observed the presence of spectral features that match those in aqueous solution, further supporting this assignment. Similarly, the broad peak at 1090 cm^–1^ is assigned as the *v*_3_ (*t*_2_) asymmetric stretch vibrational mode of SO_4_^2–^ in the aqueous core of the RMs, based on previous observations of this mode, ranging from 1110 to 1099 cm^–1^ in aqueous solution.^60–62^ While the band is slightly red-shifted relative to previous observations for SO_4_^2–^, the location, blue-shifted relative to the corresponding mode of HSO_4_^–^, remains consistent. The wide range of frequencies attributed to this mode highlights the environmental sensitivity of SO_4_^2–^. Both hydration and association with local cations have been shown to shift the asymmetric stretch band of SO_4_^2–^, so it is not implausible to assume that similar variations in local environment within the RM aqueous core could be responsible for the observed frequency shift.

**Figure 2:**
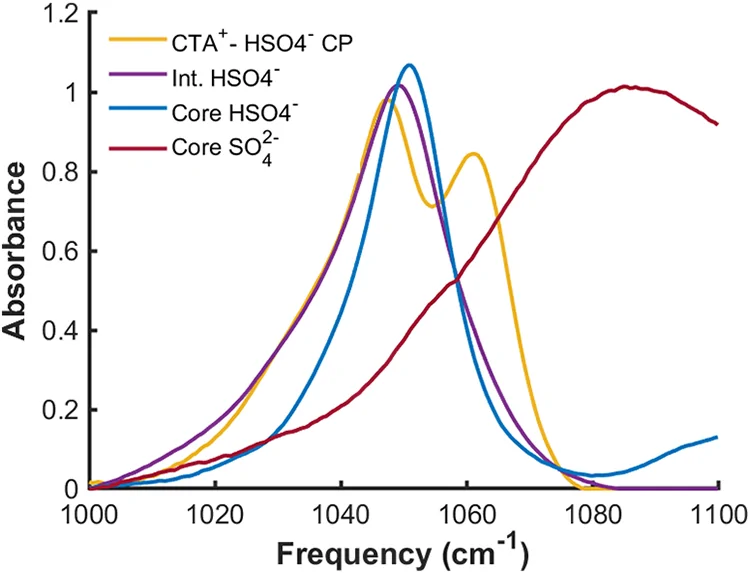
Isolated IR absorption spectra of CTA^+^-HSO_4_^–^ CP (yellow line), interfacial HSO_4_^–^ (purple line), core HSO_4_^–^ (blue line), and core SO_4_^2–^ (red line) within CTAHS RMs.

While the assignment of bands at 1051 and 1099 cm^–1^ to HSO_4_^–^ and SO_4_^2–^ in the aqueous core of the RMs can be assisted by observations in aqueous solution, developing spectral assignments for chemical species at interfaces often relies on a combination of spectral shifts and dynamic information. Interfacial water within RMs has frequently been characterized as having substantially slower reorientation dynamics and vibrational lifetimes compared to bulk water.^35–41,63^ As a result, numerous molecules located in the interfacial region have been observed to have narrower vibrational line widths or longer fluorescent lifetimes compared to their bulk solution counterparts, due to the altered dynamics within the interfacial region.^33,63–65^ In addition to altered dynamics, molecules in the interfacial region also exhibit spectral shifts relative to their spectra in bulk solution, correlated with the charge properties of the interface and the orientation of interfacial water.^33,35–41,43,63–66^ Shifts in the OD- and OH-stretches of HOD have been observed in response to charge-identity of the surfactant head, red-shifting in dioctyl sodium sulfosuccinate (AOT) RMs and blue-shifting in CTAB RMs.^35–41,43,63^ Ionic dopants have exhibited similar responses, with the NO-stretch of interfacial NO_3_^–^ in AOT RMs shifted 23 cm^–1^ to the blue of the NO-stretch in aqueous solution, due to electrostatic repulsion between the anionic SO_3_^–^ headgroup of the AOT and NO_3_^–^.^33^ Based on the spectral shift criteria, the band at 1049 cm^–1^ is likely an interfacial version of HSO_4_^–^. The similarity in band shape and location relative to aqueous HSO_4_^–^ supports the assignment of a closely related species. Moreover, the red-shift is consistent with the electrostatic interactions between the cationic trimethylammonium headgroup of CTA^+^ and the anionic HSO_4_^–^, with the attraction between the ions elongating the S–O bonds on the SO_3_ moiety of HSO_4_^–^, resulting in a lowering of the vibrational frequency of the υ_1_(*a*_1_)*v*_s_SO_3_ mode. A similar frequency of 1047 cm^–1^ for the υ_1_(*a*_1_)*v*_s_SO_3_ mode was observed in saturated NH_4_HSO_4_ solution, where the high ionic concentration would support close interactions between the HSO_4_^–^ and NH_4_^+^, but it was located at 1052 cm^–1^ for lower NH_4_HSO_4_ concentrations, where the ionic interactions were decreased.^61,67^ Vibrational sum frequency generation studies of H_2_SO_4_ solutions at air–water interfaces, similarly, identified the υ_1_(*a*_1_)*v*_s_SO_3_ mode of interfacial HSO_4_^–^ at 1050 cm^–1^.^27^ Gas-phase HSO_4_^–^(H_2_O)_*n* = 1–16_ cluster experiments observed an average frequency of 1049 cm^–1^ for *n* = 1–12 (H_2_O)_n_ clusters for the same mode.^68^ Computational calculations for HSO_4_^–^(H_2_O)_*n* = 0–6_ clusters observed a modest blue-shift as the number of H_2_O within the clusters increased, but noted the general insensitivity of the υ_1_(*a*_1_)*v*_s_SO_3_ mode to hydration number.^69^

While the assignment of the band at 1049 cm^–1^ to interfacial HSO_4_^–^ is well supported by the spectral shift criteria, the line width appears inconsistent with an interfacial assignment. The band at 1049 cm^–1^ is notably broader than that of aqueous HSO_4_^–^, 23 cm^–1^ compared to 16 cm^–1^ at fwhm, respectively. However, the broadening appears to be localized almost exclusively on the red edge of the band. The asymmetry of the band suggests the presence of some underlying feature on the red edge of the band. The bimodal spectrum, with bands located at 1047 and 1061 cm^–1^, notably overlaps with the red edge of the interfacial HSO_4_^–^ band, suggesting a close relationship between the species responsible for these spectra. To investigate the relationship between interfacial HSO_4_^–^ and species associated with the bimodal spectrum, CTA^+^ and HSO_4_^–^ were optimized at the MP2/6-311+G­(d,p) level of theory, introduced to the same system and optimized in implicit H_2_O solvent (Figure 3A). When optimized, the SO_3_ moiety is oriented toward the headgroup of CTA^+^ and separated by 3.57320 Å, with a vibrational frequency for the υ_1_(*a*_1_) *v*_*s*_ *SO*_3_ mode of 1034.62 cm^–1^. Further calculations were carried out by changing the distance between CTA^+^ and HSO_4_^–^ from the optimal distance, increasing the distance in increments of 0.5 Å up to 7.0732 Å and decreasing the distance in increments of 0.25 Å down to 2.5732 Å. Increasing the distance between the ions has a small effect on the vibrational frequency, decreasing to 1033.29 cm^–1^ at 4.0732 Å and then remaining nearly constant at approximately 1033 cm^–1^ for all larger distances. While increasing the distance has little effect on the vibrational frequency, decreasing the distance results in a large blue-shift, increasing to 1046.05 cm^–1^ at 2.5732 Å. The experimentally observed band at 1061 cm^–1^ is similarly blue-shifted relative to the core HSO_4_^–^ at 1051 cm^–1^, suggesting that this band corresponds to HSO_4_^–^ in close proximity to the CTA^+^ headgroup. However, the deconvolved spectrum consists of two bands rather than a single band, indicating a possible second HSO_4_^–^ species not represented by this set of calculations. To address the possibility of another HSO_4_^–^ species, we conducted a second series of calculations in which we rotated the HSO_4_^–^ by 180° to orient the OH moiety toward the CTA^+^ headgroup, rather than the SO_3_^–^ group in our previous calculations (Figure 3B). Vibrational frequencies were then recalculated for this “flipped” orientation at the same distances used for the “normal” orientation (SO_3_^–^ moiety oriented toward the CTA^+^). Calculated vibrational frequencies for both the normal and flipped orientations as a function of CTA^+^-HSO_4_^–^ distance are shown in Figure 4. At the optimal distance of 3.57320 Å, the vibrational frequency of the flipped HSO_4_^–^ species is 1033.72 cm^–1^, only 0.85 cm^–1^ shifted relative to the normal orientation. Similar vibrational frequencies are observed for both orientations at larger CTA^+^-HSO_4_^–^ distances. As the ionic distance decreases, the vibrational frequency of the flipped orientation red-shifts, decreasing to 1030.25 cm^–1^ at 2.5732 Å. The flipped orientation is less sensitive to decreased ionic distance compared to the normal orientation, with a frequency shift of −3.52 cm^–1^ between the optimal and closest distance (2.5732 Å), compared to +11.43 cm^–1^ for the normal orientation over the same range. The relative differences between the two bands in the bimodal spectrum and the spectrum of core HSO_4_^–^ are −4 cm^–1^ and +10 cm^–1^ for the 1047 cm^–1^ and 1061 cm^–1^ bands, respectively. The direction and relative magnitude of the calculated shifts are consistent with the experimentally observed spectral differences and provide qualitative support for assigning the band at 1047 cm^–1^ to the flipped orientation of HSO_4_^–^. Calculated IR intensities for the flipped (∼134) and normal (∼113) orientations mirror our experimental spectrum, with the band associated with the flipped orientation exhibiting a higher intensity than the normal orientation (Tables S1 and S2). Comparison of relative intensities shows additional similarities, with our calculations possessing a 0.84 normal/flipped intensity ratio compared to a 0.86 normal/flipped intensity ratio for our experimentally derived spectrum. Taken together, we assign the entire bimodal spectrum to CTA^+^-HSO_4_^–^ CP, which can be subdivided into both “normal” and “flipped” orientation subpopulations. Ionic CPs have been observed in multiple systems, including high-concentration solutions, air–water interfaces, and RMs, so our isolation of a vibration spectrum associated with CP species is consistent with these analogous systems.

**Figure 3:**
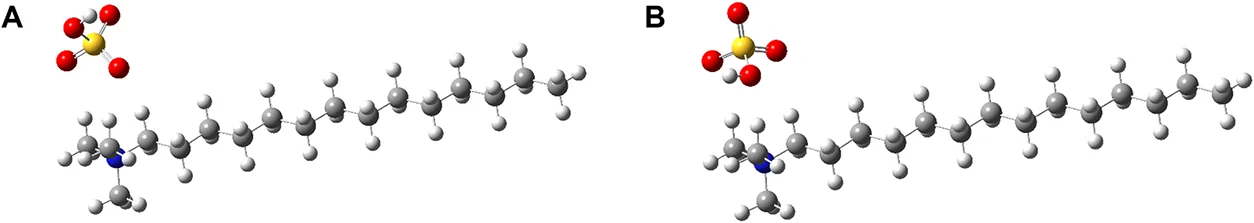
Calculated CTA^+^-HSO_4_^–^ ion pairs in the (A) “normal” orientation and (B) “flipped” orientation. Sulfur (yellow), oxygen (red), hydrogen (white), nitrogen (blue), and carbon (dark gray) atoms are displayed following the CPK coloring convention.

**Figure 4:**
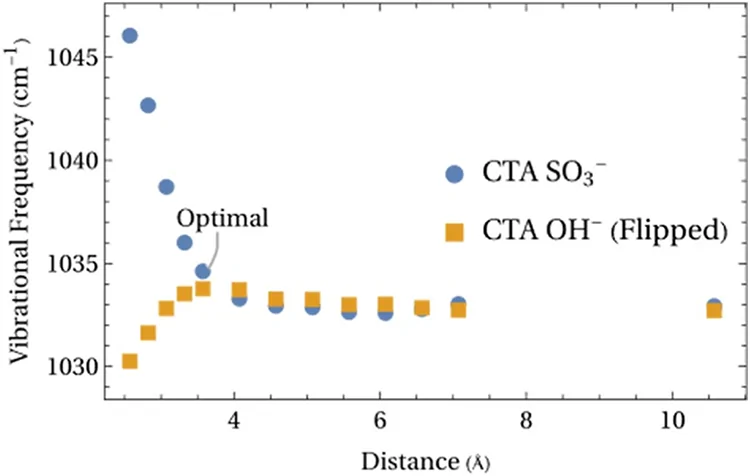
Calculated vibrational frequencies of the symmetric SO_3_ stretch of HSO_4_^–^ in CTA^+^-HSO_4_^–^ ion pairs in the “normal” and “flipped” orientations as a function of ion–ion distance in implicit H_2_O solvent using the MP2 level of theory.

Two features of this analysis bound the interpretation. The displaced structures are nonstationary points on the potential energy surface, so the calculated frequencies are not equilibrium normal modes, and their absolute values are not directly comparable with experiment. The calculated *v*_s_SO_3_ frequency at the optimized geometry, 1034.62 cm^–1^, lies roughly 16 cm^–1^ below the experimental core band at 1051 cm^–1^. We therefore base our assignments on the direction and relative magnitude of the frequency shift as the ion pair is displaced, not on an absolute agreement. The *v*_s_SO_3_ mode is well suited to this purpose, since it is a localized, high-frequency vibration of the SO_3_ moiety, far removed from the soft intermolecular coordinate along which the structures were displaced, and so comparatively insensitive to the residual gradient at a nonstationary geometry.

A single ion pair in an implicit solvent also samples one static geometry rather than the distribution of configurations present at the interface. Our comparison with the deconvolved spectrum is therefore qualitative, resting on agreement in the shift direction, relative magnitude, and intensity ordering rather than on quantitative reproduction of band positions. We think that the close match in the normal-to-flipped intensity ratio, 0.84 calculated against 0.86 experimental, is encouraging. Because these intensities were obtained from idealized ion-pair geometries rather than from a fully sampled interfacial ensemble, they should not be read as the relative populations of the two species. Nevertheless, the qualitative agreement in both frequency shifts and relative intensities supports the assignment of the bimodal spectrum to CTA^+^-HSO_4_^–^ CP species.

While ion pairs have been observed in multiple studies, sulfate species appear to be highly sensitive to the valency of the cation, forming different ion pair species, with unique orientational and dynamic properties.^29,62,70^ Studies of SO_4_^2–^ solutions noted that Mg^2+^ favored solvent shared ion pairs (SIPs), while Na^+^ formed both CPs and SIPs.^62^ The radial distributions of S–Na^+^ distances revealed that CP species maximized at 3.5 Å, similar to the N–S distances below 3.57320 Å that were expected to give rise to the distinct vibrational signatures of our “normal” and “flipped” orientations.^62^ Formation of CPs for Na^+^, but not Mg^2+^, was attributed to monovalent Na^+^ possessing a more flexible solvation shell compared to divalent Mg^2+^. Separate studies of air–water interfaces determined that formation of Mg^2+^-SO_4_^2–^ SIPs resulted in an electric field reversal in the interface, with the Mg^2+^ allocated closer to the surface than SO_4_^2–^, a marked contrast to previous studies in which anions were favored over cations at the surface.^29^

Studies focusing on HSO_4_^–^ have noted similar sensitivities to cations. Vibrational sum frequency generation experiments of air–water interfaces in the presence of Na^+^ or Mg^2+^ cations noted different vibrational frequencies for the symmetric SO_3_ stretch of HSO_4_^–^, with Mg^2+^ resulting in a single band centered at 1069 cm^–1^and Na^+^ producing a bimodal spectrum with peaks at 1048 and 1067 cm^–1^.^27^ These spectra were both shifted relative to interfacial HSO_4_^–^ in H_2_SO_4_ solutions at 1050 cm^–1^. The blue-shifted peaks were interpreted in terms of perturbations in the hydration of HSO_4_^–^ induced by the presence of the cations, rather than ion pair formation, which was expected to manifest as red-shifted spectra.^61,67^ High-concentration H_2_SO_4_ and NH_4_HSO_4_ solutions observed exclusively red-shifted spectra, relative to HSO_4_^–^ in bulk solution, ranging from 1035 to 1047 cm^–1^ for the υ_1_(*a*_1_)*v*_s_SO_3_ mode, indicating that proximity and hydrogen-bonding capacity of the cation can induce shifts in the IR spectrum of HSO_4_^–^.^67,71–73^ Valency of the cation also appears to play a role, as red-shifted spectra are primarily observed in the presence of monovalent cations and blue-shifted spectra in the presence of divalent Mg^2+^.^27^ Similar trends, albeit with more substantial frequency shifts, were observed for Na_2_SO_4_ solutions, where increasing MgCl_2_ concentrations resulted in decreases in absorbance at 1100 cm^–1^ and the corresponding increases at 1150 cm^–1^, suggesting that the Na^+^-SO_4_^2–^ ion pairs have red-shifted spectra relative to Mg^2+^-SO_4_^2–^ ion pairs.^62^

Our studies suggest that the bimodal spectra may also be related to orientational subpopulations of HSO_4_^–^ relative to the interface, in addition to hydration effects. MD simulations of H_2_SO_4_ solutions revealed that HSO_4_^–^ at the air–water interface of H_2_SO_4_ solutions was oriented with the OH group toward the air–water surface, placing the SO_3_^–^ moiety toward the bulk.^28^ This preferential orientation was interpreted in terms of maximizing hydrogen-bonding, with the oxygens on the SO_3_^–^ moiety being better hydrogen-bond acceptors than the oxygen of the OH group. As bimodal spectra for the υ_1_(*a*_1_)*v*_s_SO_3_ mode of HSO_4_^–^ have been observed at interfaces (CTAHS RMs and air–water^27^), in the presence of monovalent cations (CTA^+^ and Na^+^), with minimal or no hydrogen-bonding capacity, there appears to be a convolved or competing relationship between hydrogen-bonding/hydration effects and electrostatics in terms of directing molecular orientation in the interface. In our case, the charge of cationic CTA^+^ interface may increase competing effects between electrostatics and hydrogen-bonding/hydration within the interface compared to air–water interfaces. CPs in the “normal” orientation would provide better electrostatic balance to the CTA^+^ headgroups, while the “flipped” orientation would maximize hydrogen-bonding interactions, creating a stronger solvation shell. These observations seem to agree with the conclusions reached from studies of air–water interfaces under the application of external electric fields that ionic distributions at interfaces may be driven by both electrostatics and preferential solvation effects.^74^

Our calculations, along with the assignments within the CTA^+^-HSO_4_^–^ CP spectrum, also help reconcile the broader line width observed for interfacial HSO_4_^–^ relative to core HSO_4_^–^ with the expected vibrational lifetimes of core and interfacial species. The conversion of CTA^+^-HSO_4_^–^ CP to interfacial HSO_4_^–^ should correspond to an increase in distance between CTA^+^ and HSO_4_^–^. As ionic distance increases, the vibrational frequency of the “flipped” orientation blue-shifts, while the “normal” orientation HSO_4_^–^ red-shifts, bringing both vibrations closer in frequency to one another. As such, the breadth of the interfacial HSO_4_^–^ band appears to be a result of heterogeneous line broadening caused by the two spectrally distinct orientational subpopulations of HSO_4_^–^. As the distance between CTA^+^ and HSO_4_^–^ continues to increase, the orientation of HSO_4_^–^ relative to CTA^+^ no longer exerts any influence on vibrational frequency, eliminating any broadening effects induced by orientational heterogeneity.

Beyond determining the pure spectral components, MCR-ALS analysis also calculates the contributions of the pure spectrum to each mixed spectrum within the data set.^44,45^ As spectral and concentration determinations are calculated simultaneously, MCR-ALS analysis makes no assumptions regarding how spectral components should vary within the data set unless specified. In our case, a single closure constraint was placed on the data set, setting overall spectral partitioning to 1, to reflect that sulfate species partitioning could vary between spectra, but total sulfate concentrations should not. The partitioning between sulfate species as a function of RM *w*_0_ ratio is displayed in Figure 5 (Table S3). CTA^+^-HSO_4_^–^ CP is the dominant sulfate species at *w*_0_ = 2, corresponding to 0.48 of all sulfate species, but decays rapidly as *w*_0_ increases, reaching 0 by *w*_0_ = 10. The decrease in partitioning of CP is accompanied by an increase in the proportion of interfacial HSO_4_^–^ up to a maximum of 0.61 at *w*_0_ = 5 before decaying to 0.27 by *w*_0_ = 30. As both CP and interfacial HSO_4_^–^ decay, core HSO_4_^–^ increases in proportion, from 0.13 at *w*_0_ = 2, becoming the dominant sulfate species after *w*_0_ = 10. Core SO_4_^2–^ shows much slower growth, appearing at *w*_0_ = 8 and increasing to 0.17 by *w*_0_ = 30. The decay of interfacial species partitioning and the corresponding growth of core species as *w*_0_ increases have been previously observed experimentally and computationally for both interior H_2_O and ionic dopants in RM systems.^33,35,39–41,75,76^ The observed variation in sulfate partitioning as a function of *w*_0_ adds validity to our spectral assignments as both interfacial species, CP and interfacial HSO_4_^–^, decay as *w*_0_ increases and core species, HSO_4_^–^ and SO_4_^2–^, increase as *w*_0_ increases.

**Figure 5:**
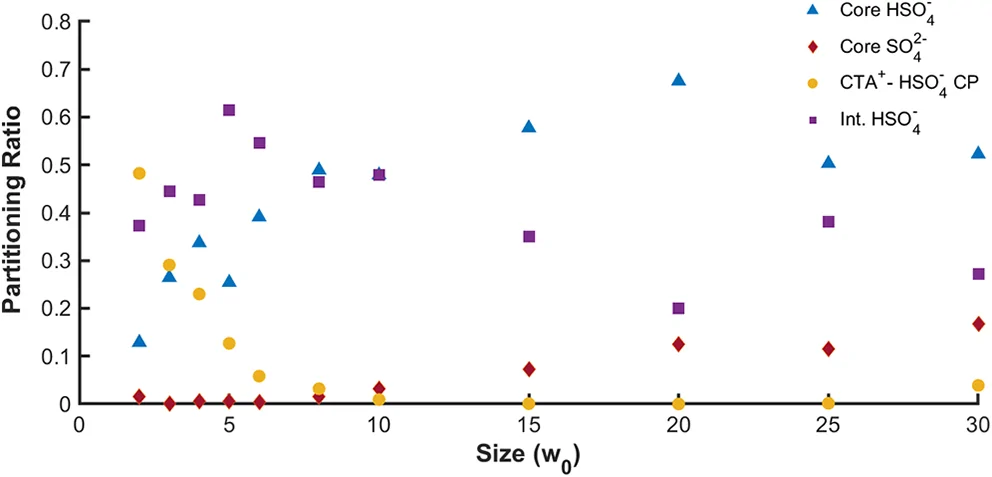
Partitioning of CTA^+^-HSO_4_^–^ CP (yellow circles), interfacial HSO_4_^–^ (purple squares), core HSO_4_^–^ (blue triangles), and core SO_4_^2–^ (red diamonds) within CTAHS RMs as a function of RM size (*w*_0_).

Previous studies have shown that interfacial partitioning (*P*_Interfacial_) as a function of *w*_0_ can be characterized by a power law of the form:

1 $$P_{Int\mathrm{erf}acial}=A{\left(w_{0}\right)}^{\left(\chi_{Int.}-1\right)}$$

where χ_Int._ is defined as the interfacial affinity.^33^ In this framework, interfacial affinity is placed on a scale of 1.0 to 0, with the values representing the highest and lowest possible interfacial affinities, respectively. In the case of HSO_4_^–^ in CTAHS RMs, we determined overall interfacial partitioning by combining the proportions of both CTA^+^-HSO_4_^–^ CPs and interfacial HSO_4_^–^, as both are present within the interfacial region of the RMs. The total interfacial partitioning as a function of *w*_0_ is shown in Figure 6. The corresponding χ_Int._ = 0.56 ± 0.03 and *A* = 1.25 ± 0.07. Previous studies determined that χ_Int._ = 0.46 ± 0.05 for NO_3_^–^ doped within anionic AOT RMs.^33^ As expected, the favorable electrostatics between CTA^+^ and HSO_4_^–^ results in a higher χ_Int._ than the repulsive charges of the SO_3_^–^ headgroups of AOT and NO_3_^–^.

**Figure 6:**
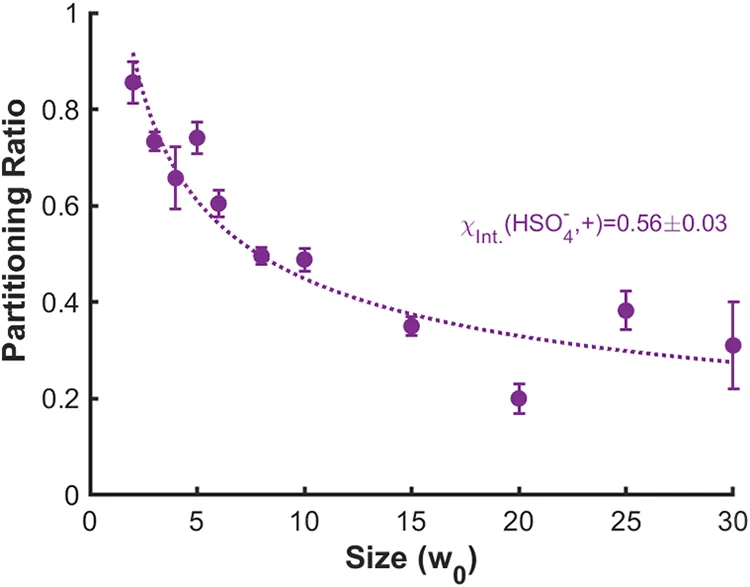
Total interfacial partitioning of HSO_4_^–^ within CTAHS RMs based on the combined partitioning ratios of CTA^+^-HSO_4_^–^ CPs and interfacial HSO_4_^–^. The interfacial partitioning curve is fit to eq 1.

Interfacial sulfate partitioning also provides insights into how the cationic charge of the RM interface exerts an influence on the overall ionic distribution within the interface. Ionic distributions at air–water interfaces have typically been cast in terms of electric double-layer formation. In the case of H_2_SO_4_ solutions, this framework results in a (H_3_O^+^) > (HSO_4_^–^) > (SO_4_^2–^) distribution in terms of preference for the interface.^28,30^ The addition of cations into the system has been framed in a similar fashion, with cations localizing between the HSO_4_^–^ and SO_4_^2–^ in the interface. As the proportion of core water within the aqueous interior increases, we see the emergence of core HSO_4_^–^ and SO_4_^2–^. The observation of SO_4_^2–^ only after *w*_0_ = 8 is consistent with the aversion of SO_4_^2–^ for the interface. The presence of core SO_4_^2–^ also suggests that H_3_O^+^ must be present within the aqueous interior. MD simulations of RM ionic distributions have suggested that multiple ionic layers exist within the RM aqueous interior.^75,76^ In the case of KCl-doped AOT RMs, multiple cationic layers containing Na^+^ and K^+^ were localized between the SO_3_^–^ headgroups at the interface and the first anionic layer of the Cl^–^ dopant.^75^ Assuming similar ionic distributions within the CTAHS RMs, we propose that H_3_O^+^ is likely localized between the interfacial HSO_4_^–^ and the core HSO_4_^–^ and SO_4_^2–^ in the following arrangement: Interface (CTA^+^) > (HSO_4_^–^ CP) > (Interfacial HSO_4_^–^) > (H_3_O^+^) > (Core HSO_4_^–^) = (Core SO_4_^2–^) Core. While this approach does not allow us to assess whether H_3_O^+^ is localized in the core or the interface, our proposed arrangement reconciles the stronger preference for anions in the interface caused by the cationic charges of the CTA^+^ headgroups with the observations from air–water interfaces that assign a strong surface preference for H_3_O^+^, while also maintaining electric double-layer arrangements often used to describe interfacial ion distributions.

## Conclusions

The interfacial distribution of sulfate species was examined, using CTAHS RMs as morphological proxies for marine aerosol particles. Using IR absorption spectroscopy and MCR-ALS analysis, we identified four distinct sulfate species, with unique vibrational signatures, within the aqueous interior of CTAHS RMs: CTA^+^-HSO_4_^–^ CPs, interfacial HSO_4_^–^, core HSO_4_^–^, and core SO_4_^2–^. CTA^+^-HSO_4_^–^ CPs exhibit a bimodal vibrational spectrum, which we attribute to two orientational subpopulations: a “normal” species, with the SO_3_^–^ moiety pointed toward the CTA^+^ headgroups, and a “flipped” species, with the OH group pointed toward the CTA^+^ headgroups and the SO_3_^–^ pointed toward the aqueous core. The unexpected breadth of the interfacial HSO_4_^–^ band, relative to the core HSO_4_^–^ vibrational band, is described in terms of heterogeneous broadening induced by the “normal” and “flipped” orientations maintaining distinct vibrational transitions over a 1 Å distance from the CTA^+^ headgroups. The breadth and asymmetry of the interfacial HSO_4_^–^ band, relative to the core HSO_4_^–^ band, are consistent with heterogeneous broadening arising from overlapping contact-pair contributions. Model calculations indicate that the normal and flipped CTA^+^–HSO_4_^–^ orientations exhibit distinct vibrational shifts as the ion-pair separation changes near the CTA^+^ headgroup. Because these calculations were performed using displaced, nonequilibrium geometries, the computed frequencies are interpreted as qualitative support for the direction and magnitude of the observed spectral shifts rather than as a complete description of equilibrium contact-pair populations. Together, the experimental and computational results support assignment of the bimodal spectrum to CTA^+^–HSO_4_^–^ CPs with distinct orientational subpopulations. With sulfate species partitioning as a function of RM, *w*_0_ ratio shows substantial evolution between sulfate species, favoring interfacial species, CTA^+^-HSO_4_^–^ CPs and interfacial HSO_4_^–^, at small *w*_0_’s and core species, core HSO_4_^–^ and SO_4_^2–^, at larger *w*_0_’s. Quantified interfacial affinity (χ_Int._) was determined by fitting the sum of the interfacial species partitioning as a function of *w*_0_ to a power law, resulting in a χ_Int._ = 0.56 ± 0.03. This parameter provides a quantitative means of describing preference for the interface. The interfacial affinity of HSO_4_^–^ in CTAHS RMs exceeds the interfacial affinity of NO_3_^–^ in AOT RMs, consistent with the more favorable electrostatics of the HSO_4_^–^ paired with a cationic CTA^+^ interface, compared with NO_3_^–^ in the presence of a repulsive anionic SO_3_^–^ interface.^33^ However, the difference between the interfacial affinities is fairly small, suggesting that electrostatics alone may not direct ionic distributions at interfaces. Experiments are currently underway to further elucidate the properties that influence ionic distributions within the interface. The quantified interfacial affinity of HSO_4_^–^ provides additional rationale for the observed substrate dependence on the heterogeneous uptake of N_2_O_5_.^1^ While sulfate species do not participate directly in the hydrolysis of N_2_O_5_, the stronger interfacial affinity of HSO_4_^–^ relative to SO_4_^2–^ results in the displacement of H_2_O from the interface, slowing the hydrolysis of N_2_O_5_. Further quantification of the interfacial affinities of ions could help provide molecular-scale details regarding ionic distributions and assist in describing heterogeneous reaction mechanisms.

## Supplementary Material





## Notes

IR absorption spectra of *w*_0_ = 2 CTAHS RM and corresponding fit using scaled pure component spectra (Figure S1); IR absorption spectra of *w*_0_ = 5 CTAHS RM and corresponding fit using scaled pure component spectra (Figure S2); IR absorption spectra of *w*_0_ = 10 CTAHS RM and corresponding fit using scaled pure component spectra (Figure S3); and IR absorption spectra of *w*_0_ = 20 CTAHS RM and corresponding fit using scaled pure component spectra (Figure S4) (PDF)
Vibrational frequencies of CTA^+^-HSO_4_^–^ ion pairs in normal configuration (Table S1); vibrational frequencies of CTA^+^-HSO_4_^–^ ion pairs in flipped configuration (Table S2); and partitioning ratios of sulfate species in CTAHS RMs (Table S3) (PDF)
