# Supplementary material for: Quantified Interfacial Affinity of Hydrogen Sulfate and Sulfate Speciation in Model Marine Aerosol Particles
Source: J Phys Chem A. 2026 Sep 4;130(37):7460–8. doi: 10.1021/acs.jpca.6c04034 (PMC13592070; doi:10.1021/acs.jpca.6c04034)
Supplement: Supplementary file 2 [file jp6c04034_si_002.pdf]

Supporting Information:

Quantified Interfacial Affinity of Hydrogen Sulfate and Sulfate Speciation in Model Marine Aerosol Particles

Leslie M. Ritchie[†], *Madison B. Lester*[†], *Arun K. Sharma*[‡], *Joshua D. Patterson*[*,†]

[†]Department Biology, Chemistry, and Environmental Science, Christopher Newport University, Newport News, Virginia 23606, United States

[‡]Department of Biology, Agriculture, and Chemistry, California State University Monterey Bay, Seaside, California 93955, United States

Table S1. Vibrational Frequencies of CTA^{+}-HSO_4^{-} Ion Pairs in Normal Configuration

Calculation	Distance	Frequency	Infrared	Energy
Freqin4	2.5732 Å	1046.05 /cm	113.924 /cm	-951.533 E_h
Freqin3	2.8232 Å	1042.67 /cm	160.545 /cm	-951.587 E_h
Freqin2	3.0732 Å	1038.71 /cm	164.968 /cm	-951.612 E_h
Freqin1	3.3232 Å	1036.01 /cm	160.009 /cm	-951.622 E_h
OptFreq	3.5732 Å	1034.62 /cm	157.132 /cm	-951.624 E_h
Freq1	4.0732 Å	1033.29 /cm	151.482 /cm	-951.621 E_h
Freq2	4.5732 Å	1032.94 /cm	147.697 /cm	-951.617 E_h
Freq3	5.0732 Å	1032.87 /cm	146.516 /cm	-951.615 E_h
Freq4	5.5732 Å	1032.64 /cm	146.245 /cm	-951.615 E_h
Freq5	6.0732 Å	1032.59 /cm	146.073 /cm	-951.614 E_h
Freq6	6.5732 Å	1032.78 /cm	145.971 /cm	-951.614 E_h
Freq7	7.0732 Å	1033.03 /cm	145.346 /cm	-951.613 E_h
Freq14	10.5732 Å	1032.93 /cm	146.129 /cm	-951.613 E_h

Table S2. Vibrational Frequencies of CTA^+-HSO_4^- Ion Pairs in Flipped Configuration

Calculation	Distance	Frequency	Infrared	Energy
Freqin4	2.5732 Å	1030.25 /cm	134.229 /cm	-951.455 E_h
Freqin3	2.8232 Å	1031.63 /cm	155.431 /cm	-951.544 E_h
Freqin2	3.0732 Å	1032.82 /cm	145.255 /cm	-951.591 E_h
Freqin1	3.3232 Å	1033.53 /cm	146.587 /cm	-951.612 E_h
OptFreq	3.5732 Å	1033.77 /cm	146.821 /cm	-951.62 E_h
Freq1	4.0732 Å	1033.72 /cm	148.078 /cm	-951.621 E_h
Freq2	4.5732 Å	1033.27 /cm	146.867 /cm	-951.618 E_h
Freq3	5.0732 Å	1033.25 /cm	145.788 /cm	-951.616 E_h
Freq4	5.5732 Å	1033. /cm	145.611 /cm	-951.615 E_h
Freq5	6.0732 Å	1033.02 /cm	145.748 /cm	-951.614 E_h
Freq6	6.5732 Å	1032.85 /cm	145.443 /cm	-951.614 E_h
Freq7	7.0732 Å	1032.74 /cm	145.251 /cm	-951.613 E_h
Freq14	10.5732 Å	1032.71 /cm	146.043 /cm	-951.613 E_h

Table S3. Partitioning Ratios of Sulfate Species in CTAHS RMs

w_0	CTA^+- HSO_4^- CP	Interfacial HSO_4^-	Core HSO_4^-	Core SO_4^{2-}
2	0.48±0.03	0.37±0.03	0.13±0.04	0.02±0.01
3	0.28±0.02	0.45±0.01	0.27±0.01	0.00±0.00
4	0.23±0.02	0.43±0.06	0.34±0.07	0.01±0.01
5	0.13±0.02	0.61±0.02	0.25±0.02	0.00±0.00
6	0.06±0.01	0.55±0.03	0.39±0.03	0.00±0.00
8	0.03±0.00	0.46±0.02	0.49±0.02	0.01±0.00
10	0.01±0.00	0.48±0.02	0.48±0.02	0.03±0.01
15	0.00±0.00	0.35±0.02	0.58±0.01	0.07±0.01
20	0.00±0.00	0.20±0.03	0.68±0.02	0.12±0.01
25	0.00±0.00	0.38±0.04	0.50±0.02	0.11±0.02
30	0.04±0.02	0.27±0.09	0.52±0.04	0.17±0.04
